# How service users and carers understand, perceive, rephrase, and communicate about “depressive episode” and “schizophrenia” diagnoses: an international participatory research

**DOI:** 10.1007/s00127-020-01836-6

**Published:** 2020-02-22

**Authors:** Jean-Luc Roelandt, Antoine Baleige, Marie Koenig, Vincent Demassiet, Mohamed Agoub, Victoria Barikova, Dalila Benmessaoud, Floriane Brunet, Mauro-Giovanni Carta, Giulio Castelpietra, David Crepaz-Keay, Nicolas Daumerie, Audrey Fontaine, Neringa Grigutyte, Jugal Kishore, Marta Kiss, Marc Laporta, Elkhansaa Layoussif, Youssouf Limane, Marcelino Lopez, Gioia Mura, Jean-François Pelletier, Mbolatiana Raharinivo, Sami Richa, Rebecca Robles-Garcia, Anne-Claire Stona, Marina Skourteli, Catherine Thévenon, Michel Triantafyllou, Fotis Vasilopoulos, Stéphanie Wooley, Geoffrey Reed, Mathilde Guernut, Shekhar Saxena, Françoise Askevis-Leherpeux

**Affiliations:** 1 EPSM Lille-Métropole, French WHO CC, Armentières, France; 2 French WHO CC, Lille- Hellemes, France; 3 Equipe Inserm, Eceve (UMR 1123), Paris, France; 4 French Hearing Voices Network, Paris, France; 5 Centre Psychiatrique Universitaire Ibn Rochd, Casablanca, Morocco; 6 Centre DAccueil Et de Soins Hospitaliers, Nanterre, France; 7 Etablissement Hospitalo-Universitaire Spécialisé de Psychiatrie Mahfoud BOUCEBCI, Chéraga, Algérie; 8 Centro di Psichiatria e Psicosomatica Azienda Ospedaliero, Universitaria di Cagliari, Cagliari, Italia; 9 Primary Care Services Area, Central Health Directorate, Regione Friuli Venezia Giulia, Trieste, Italy; 10 Mental Health Foundation, London, UK; 11 Vilnius University, Vilnius, Lithuania; 12 Safdarjung Hospital, New Delhi, India; 13 Community Psychiatry Centre of Semmelweis, Awakenings Foundation, Budapest, Hungary; 14 Department of Psychiatry, McGill University, WHO CC, Montreal, Canada; 15 Ministère de la Santé, Nouakchott, Mauritanie; 16 Fundación Pública Andaluza para la Integración Social de Personas con Enfermedad Mental (FAISEM), Seville, Spain; 17 Centre de Recherche de l’Institut Universitaire en Santé Mentale de Montréal, Montréal, Canada; 18 Ministère de la Santé Publique, Tananarive, Madagascar; 19 Hôpital Hôtel de Dieu de France, Beyrouth, Lebanon; 20 Instituto National de Psiquatria “Ramón de la Fuente Muñiz”, Mexico, Mexico; 21 Association for Regional Development and Mental Health (EPAPSY), Athens, Greece; 22 Mental Health Europe, Bruxelles, Belgium; 23 Department of Mental Health and Substance Abuse, World Mental Health Organization, Geneva, Switzerland

**Keywords:** International Classification of Diseases, Clinical utility, Communication, Participatory research, Service users, Carers

## Abstract

**Background:**

For ICD-11, the WHO emphasized the clinical utility of communication and the need to involve service users and carers in the revision process.

**Aims:**

The objective was to assess whether medical vocabulary was accessible, which kinds of feelings it activated, whether and how users and carers would like to rephrase terms, and whether they used diagnosis to talk about mental health experiences.

**Method:**

An innovative protocol focused on two diagnoses (depressive episode and schizophrenia) was implemented in 15 different countries. The same issues were discussed with users and carers: understanding, feelings, rephrasing, and communication.

**Results:**

Most participants reported understanding the diagnoses, but associated them with negative feelings. While the negativity of “depressive episode” mostly came from the concept itself, that of “schizophrenia” was largely based on its social impact and stigmatization associated with “mental illness”.

When rephrasing “depressive episode”, a majority kept the root “depress*”, and suppressed the temporal dimension or renamed it. Almost no one suggested a reformulation based on “schizophrenia”. Finally, when communicating, no one used the phrase “depressive episode”. Some participants used words based on “depress”, but no one mentioned “episode”. Very few used “schizophrenia”.

**Conclusion:**

Data revealed a gap between concepts and emotional and cognitive experiences. Both professional and experiential language and knowledge have to be considered as complementary. Consequently, the ICD should be co-constructed by professionals, service users, and carers. It should take the emotional component of language, and the diversity of linguistic and cultural contexts, into account.

## Introduction

Twenty-seven years after the tenth edition of the International Classification of Diseases (ICD 10, 1992), the 11th version was adopted on May 25, 2019, and will come into effect in January 2022. Future updates should be performed every 1–10 years.

While ICD-10 was primarily aimed at professionals, the World Health Organization (WHO) emphasized the clinical utility of the classification as one of the main goals of ICD-11 [1]. As clinical utility is based on communication, the WHO decided for the first time to involve all stakeholders in the revision process, including users of mental health services and carers [2].

This paper illustrates this process in the mental health field.

### The case of “mental and behavioral disorders”

Mental health service users and carers are recognized as “experts by experience” on an international level and this has become one of the guiding principles of mental health policies [3]. As such, participatory research has now become an integral part of the methodology used in health care settings.

Moreover, access to information is considered one of the key dimensions of empowerment for mental health service users and carers [4]. For many years now, providing information has also been recognized as an important therapeutic tool [5]. Finally, it is an ethical and legal prerequisite to the user’s decision—through expression of consent—and respect for the person’s dignity and autonomy [6]. Taking users’ experience of their diagnoses into account should improve the scientific basis and accuracy of diagnoses and communication. As practice and research become more inclusive, professionals need to adopt a language which respects human rights and avoids reductive, labeling, or stigmatizing terms [7]. The changes in naming also reflect changes in medical practice, from an asymmetrical and often paternalistic relationship to a true partnership, as illustrated by the Montreal model of patient partnership [8], and the French citizenship psychiatry movement [9].

Finally, effective communication requires that words and phrasings are accessible and understandable by everyone. Because the ICD is an international standard, and language is not free from culture, the naming of category labels has to be questioned [10]. A direct translation from the English denomination might not be the most appropriate term and phrasing should be discussed with all stakeholders, especially users and carers. Effective communication also entails that the underlying concept exists on both sides, independent of culture or organization of mental health services.

### Background

The French WHO Collaborating Center (WHO-CC, in Lille), in collaboration with the WHO Geneva, and the Medical, Scientific, Health, Mental Health and Society Research Centre (CERMES) organized meetings on the revision process, with the active participation of French speaking professionals and the French Federation of Psychiatry [3]. These meetings of clinicians, researchers, users, and carers led to the creation of a French-speaking consortium to support the WHO in the revision process of the diagnostic classification system.

It was decided that the Canadian WHO-CC (Montreal) would focus on the revision of current Z-coded contextual factors, and the French WHO-CC would focus on F-coded diagnoses and their essential features on an international level.

A first meeting, bringing together all partners, was held prior to the study (Summer 2016), to agree on a consensual international protocol, develop shared materials, train investigators, and discuss translations. A second meeting took place after the end of data collection and analyses (Spring 2018), to discuss and interpret the results, and to agree on recommendations to be addressed to the WHO.

### A focus on “depressive episode” and “schizophrenia”

Even if the diagnoses are different in terms of prevalence, impact, and social representations [11], both are stigmatizing, which can limit access to information and care [12].

“Depression” is now recognized by the WHO as the most common diagnosis of mental disorder and the leading cause of disability worldwide [13]. Although effective treatments exist, fewer than half of those supposedly affected in the world (in some countries, less than 10%) benefit from those treatments [14], mainly due to a lack of information and negative stereotypes associated with the diagnosis [15]. There is also a lack of consistency in the methods of assessment and the resulting diagnoses, which may have a direct impact on a person’s health, either because of the risks associated with unnecessary treatment due to over-diagnosis, or because of the lack of access to care due to under-diagnosis.

“Schizophrenia” is considered the fifth largest disabling diagnosis of mental disorders in the world [13] and the most stigmatizing psychiatric label across countries [16], linked to its historic construction [17]. It is now recognized that carers for people with a diagnosis of “schizophrenia” are also highly impacted, psychologically, socially, and economically [18]. Communication about the diagnosis is a major issue. First, the experience of distress may make it difficult for some users to access information [19]. Second, social stigmatization makes some professionals reluctant to announce this diagnosis [20]. These attitudes contribute to increase the communication gap among and between professionals, users, and carers [21], and to reduce the apparent incidence of “schizophrenia” [22]. To overcome these difficulties, the Japanese Society of Psychiatry and Neurology decided to replace the original term, “Seishin-Burentsu-Byo” (in English: mind-split disease), with a new one, “Togo-Shitcho-Sho” (in English: integration disorders). This change almost doubled the percentage of diagnoses in 3 years (from 36.7 to 69.7%) and reduced stigmatization [23]. Some associations and researchers go beyond mere phrasing matters, and argue that “schizophrenia does not exist” and that the concept might be replaced by “psychosis spectrum disorders” [24].

### Objectives

The objectives were to assess whether users and carers:

1. Report understanding the diagnosis of “depressive episode” or “schizophrenia”,
2. Associate it with a negative feeling, and, if so, why?
3. Would like to rephrase it, why, and using which terms?
4. Use the diagnosis label when they talk with family and friends about their own (users), or the person they care for (carers), mental health condition.

The first three were extended to essential features of both diagnoses [25].

Results were communicated to the WHO. They were used in making modifications to the final diagnostic guidelines, especially for schizophrenia. These results were used to make the diagnostic guidelines more understandable, less stigmatizing, use more culturally relevant language in descriptions of categories, and to help translations.

## Methods

At the request of the WHO, the French WHO-CC designed an innovative protocol assessing whether ICD vocabulary is accessible, which kinds of feelings it evokes, whether and how users and carers would like to rephrase terms, and whether they use the diagnosis to talk about mental health conditions.

The protocol was implemented from August 2016 to March 2018 in 15 countries (20 research sites): Algeria, Canada, France, Greece, Hungary, India, Italy, Lebanon, Lithuania, Madagascar, Mauritania, Mexico, Morocco, Spain, and UK.

### Participation of persons with lived experience

Users and carers were involved in all the stages of the project [26]: they participated in the development of the protocol, in the review of the design and the materials, in pretesting, and in the interpretation of the results.

### Participants and design

Users were recruited as they visited the mental health services located at the research study sites, with the following inclusion criteria. They needed to have been diagnosed with “depressive episode” or “schizophrenia”, and to have attended an outpatient health service.

Carers had to provide assistance to a mental service user, either on a part-time or full-time basis, on a personal basis, and in a continuous manner. As such, professional carers were not included. Carers had to know the diagnosis and were contacted with the consent of the user for whom they were caring. Carers were not necessarily caring for the users who were included in the study (43% for depressive episode, 64% for schizophrenia), either because users did not have a carer, or because carers did not know the diagnosis or did not consent to participate. Consequently, users’ and carers’ answers were treated as independent samples.

Interviewers had to be health professionals working in the field of mental health, including peer workers and experts by experience.

### Ethical issues

The protocol complied with the Helsinki Declaration of 1975, as revised in 2008, and the WHO Good Clinical Research Practice (GCRP) guidelines, and each site submitted the protocol for agreement to the local or national relevant authorities. Informed written consent was obtained from every participant.

According to French law, the WHO-CC of Lille received agreement from relevant authorities to upload, store, and analyze databases. The data protection and management procedures were approved by specific regulations in force in France (i.e. the sponsor country).

### Procedure

The study was based on face-to-face questionnaires, focused on either “depressive episode” or “schizophrenia”. The questionnaires for users and carers were organized in the same manner, with only some questions specific to one group (users vs. carers). In such cases, users answered for themselves, and carers for the user they cared for.

Questionnaires were composed of two main sections: background information, including socio-demographic data (gender; age; marital status; way of life; family background; occupation; level of education) and a package of four questions about the phrase “depressive episode” or the word “schizophrenia”.

1. *Understanding* Do you understand the phrase (or the word)?
2. *Feelings* What does this phrase (or this word) bring to mind? [possible answers: something positive—negative or—neither]. For each answer, explain why?
3. *Rephrasing* Would you rephrase this phrase (or this word) and why? [possible answers: it is clearer, more understandable—it better matches the current or past lived experience—it is more positive (multiple choices possible)]. If rephrasing, in which term(s)?
4. *Communication* Can you talk to your family and friends about your (or that of the person you care for) mental health? If yes, what terms do you use?

Each country translated materials from original French and English documents, using a back–forward procedure, and encoded data in one of those two languages.

Overall samples were composed of 279 users and 232 carers for “depressive episode”, and of 263 users and 255 carers for “schizophrenia”.

### Analyses of verbal materials

- Propositions of rephrasing were classified by two independent coders, with the help of a third one in case of disagreement, into three categories: (1) keeping the etymological roots of the diagnosis: “episod*” and “depress*”for “depressive episode”; “*schizo*” and “*phren*” for “schizophrenia”; (2) using essential features of the diagnosis, either exact or derived words (3) neither type of classification.
- Analysis of terms used to communicate with relatives focused on the first category.
- Motivations of negative feelings were analyzed using the “Iramuteq” quali-quantitative software. Iramuteq, based on hierarchical descending classification [27], allows the extraction of differentiated categories from textual data and the exploration of whether external variables (here: status as user or carer) are associated with them. Categories were labeled as a function of their most representative elements and the context of their occurrence.

### Statistical analyses

Means and standard deviations (SDs) were calculated for continuous variables, and frequency (in percentages) for categorical variables. Comparisons between users and carers were based on Student *t* or *χ*^2^ tests, according to the type of variables contrasted (continuous or categorical, respectively).

Data were analyzed with Statistica version 10.

## Results

Results concerning motivations of feelings, reformulations, and communication are illustrated by quotations of responses from participants worldwide (Appendix 1).

### Population

In line with WHO statistics, a majority of users diagnosed with “depressive episode” were females, and inversely for “schizophrenia” (Table 1).

**Table 1:** Socio-demographic characteristics of service users and carers for the diagnoses of depressive episode and schizophrenia

Status	Users	Carers	Statistics
Total samples	*n* = 279	*n* = 232	
Depressive episode			
Gender: women	191 (68.9%)	139 (59.9%)	*χ*^2^_=_ 4.5, *df* = 1, *p* = 0.03
Age	45 ( 14.6,18–84)	44.6 (15.1, 18–82)	*t* = 0.3, *df* = 507, *p* = 0.78
Marital status: single	91 (32.6%)	56 (24.2%)	*χ*^2^_=_ 4.3, *df* = 1, *p* = 0.04
Way of life: alone	53 (19.1%)	19 (8.2%)	*χ*^2^_=_ 12.3, *df* = 1, *p* < 0001
Family: no child	101 (36.6%)	71 (30.7%)	*χ*^2^_=_ 1.9, *df* = 1, *p* = 0.16
Occupation: yes	122 (45.2%)	130 (56.5%)	*χ*^2^_=_ 7.6, *df* = 1, *p* = 0.005
Age at the end of studies^a^	*n* = 244 21.2 (6.5, 8–52)	*n* = 207 21 (6.7, 9–61)	*t* = 0.3, *df* = 449, *p* = 0.79

Total samples	Users *n* = 263	Carers *n* = 255	Statistics
Schizophrenia			
Gender: women	84 (32.1%)	165 (64.7%)	*χ*^2^ _=_ 55.2, *df* = 1, *p* < 0.001
Age	39.4 (12.1, 18–77)	55.1 (13.8, 18–87)	*t* = 13.8, *df* = 516, *p* < 0.001
Marital status: single	181 (68.8%)	28 (11%)	*χ*^2^ = 180, *df* = 1, *p* < 0.001
Way of life: alone	44 (16.7%)	18 (7.1%)	*χ*^2^_=_ 11.4, *df* = 1, *p* < 0.001
Family: no child	189 (72%)	31 (12.2%)	*χ*^2^ _=_ 1192, *df* = 1, *p* < 0.001
Occupation: yes	81 (31%)	110 (43.7%)	*χ*^2^ _=_ 8.7, *df* = 1, *p* = 0.003
Age at the end of studies^a^	*n* = 243 20.7 (5.72, 6–50)	*n* = 222 21 (7.2, 10–69)	*t* = 0.5, *df* = 463, *p* = 0.63

Data are *n* (%) or *m* (SD), range

^a^Based on those declaring to have been schooled

Regarding the diagnosis of “depressive episode”, post hoc analyses showed that users and carers had similar profiles, in terms of age, level of education, and family composition. Nevertheless, compared to carers, users were more often female, single, alone, and without children.

Regarding the diagnosis of “schizophrenia”, users and carers were similar in terms of level of education, but differed on other criteria. Compared to users, carers were more often female, older, more often employed and less often single, alone, and childless.

### “Depressive episode”

#### Understanding

More than 90% of users and carers said that they understood the diagnosis label. There was no difference between users and carers (Table 2).

**Table 2:** Depressive episode: understanding, negative feelings, rephrasing, and talking, as a function of status (service user vs. carer)

Total samples	Users	Carers	Statistics
	279	232	
Understanding, yes	257 (92.4%)	208 (90.8%)	*χ*^2^_=_ 0.43, *df* = 1, *p* = 0.41
Negative feelings, yes	237 (85.7%)	192 (83.8%)	*χ*^2^_=_ 0.40, *df* = 1, *p* = 0.53
Rephrasing, yes	100 (36%)	76 (32.8%)	*χ*^2^_=_ 0.58, *df* = 1, *p* = 0.45
If yes, suggested reformulations^a^			
Etymological roots	51 (46.3%)	47 (56%)	*χ*^2^ = 1.71, *df* = 1, *p* = 0.19
“Episod”	3 (2.7%)	1 (1.2%)	
“Depress”	45 (40.9%)	45 (53.6%)	
“Episod” and “depress”	3 (2.7%)	1 (1.2%)	
Other	59 (53.6%)	37 (44%)	
Essential features	13 (11.8%)	6 (7.1%)	
Neither	46 (41.8%)	31 (36.9%)	
Talking, yes	201 (72.6%)	178 (78.4%)	*χ*^2^ = 2.29, *df* = 1, *p* = 0.13
If yes, used terms^a^			
Diagnosis	0 (0%)	0 (0%)	
Etymological roots	31 (16%)	60 (35.3%)	
“Episod”	1 (0.5%)	0	
“Depress”	30 (15.5%)	60 (35.3%)	
Neither	162 (83.9%)	110 (64.7%)	

Data are *n* (%)

^a^All those who wanted rephrasing did not propose a precise reformulation and other ones proposed several possibilities; all those who reported that they could talk with relatives did not specify used terms

#### Feelings

A large majority of users and carers associated “depressive episode” with negative feelings. There was no difference between users and carers.

Analysis of feelings’ motivations identified four classes based on 320 (79.6%) data segments:

1. Class 1 (*n* = 185 [57.8%]): “negativity of depress*”.
2. Class 2 (*n* = 52 [16.2%]): associated with carers: “mental illness”.
3. Class 3 (*n* = 42 [13.1%]): “impact on life and difficulties and/or ways to cope”.
4. Class 4 (*n* = 41 [12.8%]): associated with users: “negative thoughts, experience, and memory”.

#### Rephrasing

About one-third of users and carers suggested a reformulation.

The most frequently quoted reasons for rephrasing were “clearness, understandability” (*n* = 86 [38%]), and “matching experience” (*n* = 84 [37.5%]) followed by “positivity” (*n* = 27 [12%]).

A significant part of reformulations was based on the “depress” root. A smaller proportion used essential features. Suggestions based on the root “depress*’” consisted either in suppressing “episod*” and renaming the diagnosis as “depression” or “depressive state”, or in keeping the temporal dimension using “period”, “phase”, or “crisis” instead of “episod*”. The most frequently quoted term from essential features was “hopelessness”.

#### Communication

A similar majority of users and carers said that they could talk to family and friends about their (or the person they care for) mental health condition. There was no difference between users and carers.

Among those who reported communicating with their relatives, no one used the phrase “depressive episode”.

### “Schizophrenia”

#### Understanding

A majority of users and carers reported understanding the diagnosis. This majority was higher for carers than for users (Table 3).

**Table 3:** Schizophrenia: understanding, negative feelings, rephrasing, and talking, as a function of status (service user vs. carer)

Total samples	Users	Carers	Statistics
	263	255	
Understanding, yes	168 (64.6%)	191 (76.4%)	*χ*^2^_=_ 8.49, *df* = 1, *p* = 0.004
Negative feelings, yes	192 (75.3%)	197 (79.4%)	*χ*^2^_=_ 2.43, *df* = 1, *p* = 0.12
Rephrasing, yes	106 (40.3%)	91 (35.7%)	*χ*^2^_=_ 1.17, *df* = 1, *p* = 0.28
If yes, suggested reformulations^a^			
Etymological roots	5 (4.2%)	2 (2.3%)	
“Schizo”	2 (1.7%)	0	
“Phrenia”	2 (1.7%)	0	
“Schizo” and “Phrenia”	1 (0.8%)	2 (2.3%)	
Other	113 (95.8%)	87 (97.7%)	
Essential features	17 (14.4%)	6 (6.7%)	
Neither	96 (81.4%)	81 (91%)	
Talking, yes	170 (65.6%)	193 (77.2%)	*χ*^2^ = 8.31, *df* = 1, *p* = 0.004
If yes, used terms^a^			
Diagnosis	28 (17.2%)	19 (10.4%)	
Etymological roots	1 (0.6%)	0	
“Schizo”	1 (0.6%)	0	
“Phrenia”	0	0	
Neither	134 (82.2%)	125 (89.6%)	

Data are *n*; %

^a^All those who wanted rephrasing did not propose a precise reformulation and other ones proposed several possibilities; all those who reported that they could talk with relatives did not specify used terms

#### Feelings

More than 75% of users and carers associated “schizophrenia” with negative feelings. There was no difference between users and carers.

Analysis of negative feelings’ motivations identified four classes based on 270 (77.1%) data segments:

1. Class 1 (42.2%): “social and cultural negative side effects of the diagnosis”.
2. Class 2 (40%): “mental illness”, sometimes associated with “chronicity”.
3. Class 3 (9.3%): “feeling of fear regarding the word and its image”.
4. Class 4 (8.5%): “difficulties to understand and/or handle the diagnosis”.

### Rephrasing

More than one-third of both users and carers suggested a reformulation. Reformulations were most often based on “clearness, understandability” (*n* = 113 [38.8%]), and “matching experience” (*n* = 82 [28.2%), followed by positivity (*n* = 53[18.2%]). The pattern was the same for users and carers. Almost no one kept the etymological roots of the diagnosis. A small proportion used essential features, most frequently related to modifications of thinking and behavior.

#### Communication

A majority, higher for carers than for users, said they can talk to family and friends about their (or the person they care for) mental health state condition. Carers communicated more frequently than users.

Among those who reported communicating with their relatives, only a minority of users used the term “schizophrenia”.

## Discussion

This project was based on the argument that maximizing clinical utility of ICD-11 would require the strengthening of communication between all stakeholders, and making words and phrasings accessible to everyone.

In line with this argument, an international participatory study conducted in 15 different countries and focused on “depressive episode” and “schizophrenia”, examined whether service users and carers reported understanding the diagnosis, whether they associated it with a negative feeling, whether they would prefer rephrasing it, and in which terms, and whether they used the label when talking with family and friends.

Results first confirmed the feasibility and the informativeness of the procedure. They revealed a gap between official medical language and users’ and carers’ language.

The key conclusion is that the answers of users and carers converge toward similar patterns and conclusions for both diagnoses. A majority reported understanding diagnoses and associated them with negative feelings, and about one-third proposed to rephrase them. Moreover, the fact that users and carers had an overall similar level of education suggests that disagreements with current medical language as used in ICD cannot be attributed to any mental or behavioral disorder.

However, results also revealed specific points of interest for both diagnoses.

Most people reported understanding the phrase “depressive episode”, but did not use it to communicate. When communicating and rephrasing, they often kept “depress*”, but almost never used “episode”. This may suggest that the word “episode” is rarely used when talking about depression. The decision to use “depress*”could be read through the lens of social acceptability, as far as people can identify with depression and think it is possible to recover from it [28]. Nevertheless, the diagnosis, and mostly the word “depression” itself, was associated with negative feelings, showing a match between signifier and signified. Moreover, the fact that the negativity of “depressive episode” is partially based on its status as a mental illness questions its stigmatizing potential due specifically to word choice.

In comparison with “depressive episode”, the diagnosis of “schizophrenia” was less accessible, still highly associated with negative feelings, and nevertheless used by some people to communicate. Only a minority of reformulations were based on etymological roots. Linguistic analyses showed that the negativity of the term “schizophrenia” came from two factors: its association with mental illness, and social negative side effects, including stigmatization. Given the evidence that these factors are strongly tied with mental illness [28], this points to a strong conceptual connection between schizophrenia and mental illness, which might explain its low use in rephrasing. Indeed, the medical definition of the disorder differs from its etymological meaning of “mind split”, showing that the signifier doesn’t match the signified condition.

### Limitations

This study presents some shortcomings, related to the design itself, but also due to the construction and the international character of the ICD.

First, in line with inclusion criteria, all users had their diagnosis disclosed. It implies that professionals communicated at least that one impactful piece of information. Knowing that not all people are given their diagnosis, results and their implications may not be applicable to persons not informed of their diagnosis or outside of the health care system. Moreover, the procedure sometimes raised ethical questions, especially in sites where disclosure of diagnosis was uncommon.

The second limitation is related to the fact that, when the protocol was implemented, the ICD-11 draft was only available in English, with its French translation still ongoing. Moreover, even though the official version of ICD-10 was available in six languages (Arabic, Chinese, English, French, Russian, Spanish), and was translated by national instances in another 36 non-official languages, there was no translation available in two participating sites (India and Madagascar). Finally, translating a word implies that the underlying concept exists, refers to the same elements, and is well rooted in society. It was not the case for all countries involved in the project, suggesting further analyses of their linguistic and cultural specificities. But, even if mentioned as a limitation, it supports the WHO’s decision to develop ICD-11 in more languages than its official ones, and to take cultural variations into consideration [10]. This limitation aligns with those inherent in the ICD itself and the difficulty to represent, in an international context, complex concepts with mutually shared terms.

Despite these limitations, results, including examination of verbal answers, suggest a gap between the concepts underlying diagnostics, and emotional and cognitive experiences. Medical language was not always in line with experiential knowledge and often had negative connotations, which could reduce the quality of communication. Moreover, data regarding both diagnoses suggest that rephrasing is mostly based on social and personal acceptability, and alignment with lived experience which may be incorrectly captured by medical language. This is in line with what has been described and recognized as experiential knowledge [29], and could be a source of relevant information to reduce the gap between chosen terms and underlying concepts. This would contribute to solving actual issues regarding the diagnosis of “schizophrenia” [21, 24], and potential issues for the diagnosis of “depressive episode”.

Some scholars consider medical and experiential knowledge to be related to different semantic and cultural spaces. For them, making a medical classification both accessible and minimally negative and stigmatizing entails adding to ICD-11 a lay version of diagnostic information [30]. Contrary to this point of view, our position is to consider both languages as complementary. Consequently, because communication has to be based on a shared language, we support that a medical classification has to be co-constructed by all stakeholders and take diversity of linguistic and cultural contexts into account. This approach should be in line with the transformation of mental health services to a global approach, consistent with human rights and particularly with the United Nations Convention on the Rights of People with Disabilities.

## Data Availability

Data are available upon reasonable request to the EPSM Lille–Métropole.

## Appendix 1

Quotations from verbatim for depressive episode and schizophrenia: motivations of negative feelings, rephrasing, and communication.
